# A comparison of 3-D CT and 2-D plain radiograph measurements of the wrist in extra-articular malunited fractures of the distal radius

**DOI:** 10.1177/17531934231213790

**Published:** 2023-11-21

**Authors:** Camiel J. Smees, Eline M. van Es, Gabriëlle J. M. Tuijthof, Joost W. Colaris, Feike de Graaff, Anne J. H. Vochteloo

**Affiliations:** 1 Centre for Orthopaedic Surgery and Sports Medicine, OCON, Hengelo, The Netherlands; 2 Biomedical Device Design and Production Technology, University of Twente, Enschede, The Netherlands; 3 Department of Orthopaedics and Sports Medicine, Erasmus University Medical Centre, Rotterdam, The Netherlands

**Keywords:** Radial inclination, volar tilt, palmar tilt, ulnar length, axial rotation, diagnostics, radius malunion

## Abstract

Two-dimensional (2-D) plain radiographs may be insufficient for the evaluation of distal radial malunion, as it is a three-dimensional (3-D) deformity. This study introduced a 3-D measuring method that outputs radial inclination, ulnar variance, palmar tilt and axial rotation. To this end, a standardized and clearly defined coordinate system was constructed that allowed 3-D measurements closely resembling the conventional 2-D method in 35 patients. Mean differences between 3-D and 2-D measurements in affected wrists were 1.8° for radial inclination, 0.8 mm for ulnar variance and 3.7° for palmar tilt. In addition, inter- and intra-observer reproducibility of all 3-D and 2-D measurements were good or excellent (intraclass correlation coefficient >0.75), with 3-D reproducibility always better than 2-D. Axial rotation was present in all patients with a mean of 7.9° (SD 6.9). Although the differences between 2-D and 3-D measurements were small, 3-D evaluation enables the assessment of axial rotation and brings us closer to a routine 3-D evaluation of malunion.

**Level of evidence:** III

## Introduction

Patients with symptomatic distal radial malunions can benefit from a corrective osteotomy. The evaluation and planning of corrective osteotomies are traditionally performed on two-dimensional (2-D) radiographs by measuring radial inclination (RI), ulnar variance (UV) and palmar tilt (PT). However, evidence would suggest that 2-D radiographic evaluation is incomplete, since the deformity also affects axial rotation (AR) (Athlani et al., 2020; Cirpar et al., 2011; de Muinck Keizer et al., 2017; Lee et al., 2010; Shintani et al., 2018).

The relation between conventional 2-D measurements (RI, UV and PT) and the corresponding three-dimensional (3-D) measurements have previously been studied (Athlani et al., 2020; Miyake et al., 2013). Miyake et al. (2013) made a mirrored 3-D model of the healthy side and separately superimposed the proximal and distal parts of the affected radius onto the healthy model. Subsequently, the differences between both models resulted in 3-D angles of RI, UV and PT, which were compared to bilateral differences in 2-D measurements. They concluded 3-D to be superior to 2-D due to its almost perfect inter- and intra-observer reproducibility. Athlani et al. (2020) performed measurements in affected and healthy radiuses separately by indicating landmarks on the 3-D models and comparing these to conventional 2-D measurements. They found 2-D and 3-D both to be accurate in evaluating extra-articular distal radius malunion, with 3-D appearing more accurate in UV measurements and having the benefit of measuring axial rotation.

However, Miyake et al. (2013) provided only differences between the affected and healthy wrists, thus lacking 3-D reference values of healthy and affected wrists individually; and Athlani et al. (2020) did not provide a clear definition of the coordinate system, which makes application of their method difficult. The aim of the present study was to develop a clearly defined 3-D measuring method to measure RI, UV and PT in both wrists of patients with a one-sided distal radial malunion and to determine differences in the values between this 3-D technique and conventional 2-D measurements. Furthermore, the inter- and intra-observer reproducibility of the 2-D and 3-D techniques was assessed. Finally, the 3-D method was used to determine 3-D AR to further understand the role of this variable.

## Methods

For this retrospective comparative study, approval was obtained from the board of directors of the OCON Centre for Orthopaedic Surgery, and based on national criteria, medical ethics consent was waived.

### Data acquisition

In total, 35 patients who were treated conservatively and developed a symptomatic extra-articular distal radial malunion were identified and retrospectively included. All patients were seen between September 2014 and June 2021. Patients were included if standard (posteroanterior [PA] and lateral) radiographs of the affected wrist and a computed tomography (CT) scan of both forearms were available and if they had not undergone prior surgery of the radius or ulna. Malunion of the affected wrist was verified using the criteria of Ali et al. (2018): RI ≤15°; UV ≥3 mm; and/or PT ≤−10°. In addition, patients with a palmar displacement (PT >20°) could also be included. While not being an inclusion criterion, most patients also had standard radiographs of the unaffected side. All radiographs were taken with the forearm in neutral rotation and the elbow in 90° flexion. For the lateral image, the upper arm was alongside the body while the forearm rested with the ulnar side on the detector. For the PA image, the arm was abducted 90° at the shoulder (the wrist was at shoulder level) and the forearm rested flat on the detector. The CT scans were made with the patient in the prone position with both arms overhead and with palms facing each other, which prevents potential differences in the measurements. Both the 2-D radiographs and CT scan data were extracted in Digital Imaging and Communications in Medicine (DICOM) format from the digital medical records.

### 2-D radiological evaluation

**Figure 1.**
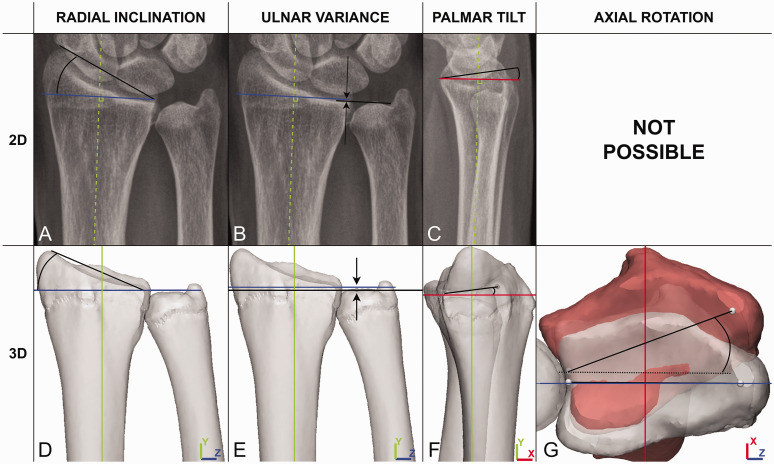
2-D and 3-D measurements of the distal radius: (a) 2-D radial inclination. (b) 2-D ulnar variance. (c) 2-D palmar tilt. (d) 3-D radial inclination. (e) 3-D ulnar variance. (f) 3-D palmar tilt and (g) 3-D axial rotation.

The 2-D radiographs were loaded into JiveX DICOM viewer (version 5.2, visus.com, Bochum, Germany, 2020). The measurements were based on the definitions proposed by Kreder et al. (1996). RI was measured on the PA radiograph as the angle between two lines: one line perpendicular to the long axis of the radius and the other line from the radial styloid process to the distal end of the distal radioulnar joint (DRUJ) surface (Figure 1(a)). UV was measured on the PA radiograph as the distance between two lines perpendicular to the long axis of the radius: one line through the distal end of the DRUJ surface and one line crossing the distal end of the ulnar dome (Figure 1(b)). PT was measured on the lateral image as the angle between two lines: one line perpendicular to the long axis of the radius and the other line through the palmar and dorsal ends of the articular surface of the distal radius (Figure 1(c)). All 2-D measurements were first performed on the affected wrists. Subsequently, the measurements were performed for the unaffected wrists. Finally, differences between both sides were calculated.

### 3-D evaluation

The CT scan data of each patient were loaded into 3-D medical imaging processing software Mimics (version 24.0, Materialise NV, Leuven, Belgium). 3-D bone surface models of the radius and ulna were created using a threshold value of 300 HU. The models were loaded into 3-D modelling software 3-Matic (version 16.0, Materialise NV, Leuven, Belgium), in which a coordinate system was defined, and 3-D measurements were performed.

To obtain clinically interpretable measurements of the malunion in 3-D, a reproducible and clinically understandable coordinate system is important, and the coordinate system should be based on an unaffected distal radius (Coburn et al., 2007; de Roo et al., 2020; Kobayashi et al., 1997; Wu et al., 2005). There are different possibilities for obtaining a reference radius, such as using the contralateral radius, a mean virtual radius (which is composed from the mean shape of a dataset of healthy radiuses) or a statistical shape model (a model that predicts normality of a bone based on the unaffected part of the bone) (Mauler et al., 2017). In this study, the contralateral radius was selected, because this is currently still considered the best reference for the affected radius. First, the unaffected radius was mirrored and superimposed over the affected radius. Superimposing the unaffected radius was done through global registration of the unaffected radius over the part of the affected radius proximal to the old fracture site. The registration was visually verified.

Second, the coordinate system was generated in accordance to the recommended definition of the International Society of Biomechanics (ISB) (Wu et al., 2005) via a series of steps:

1. The length from (proximal) the depression of the radial head to (distal) the ridge between the radioscaphoid and the radiolunate fossae was measured.
2. The radius was cut halfway, perpendicular to the long axis (Figure 2(a)).

**Figure 2.**
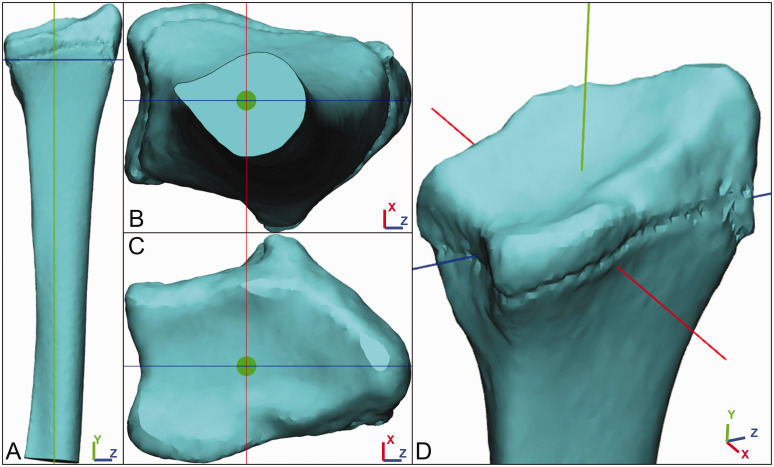
The 3-D coordinate system of the distal radius. The red line represents the X-axis, the green line the Y-axis and the blue line the Z-axis. The Y-axis is defined from the centre of the radius halfway to distally the middle of the central ridge. The Z-axis is defined perpendicular to the Y-axis from the radial styloid to the middle of the distal radioulnar joint. The X-axis is defined perpendicular to the Y- and Z-axis.
3. The proximal centre of the radius tangent to the cut plane was found (Figure 2(b), green dot).
4. The line from this proximal centre to the middle of the ridge between the radioscaphoid and the radiolunate fossae was defined. This line represents the Y-axis (Figure 2, green line).
5. A line perpendicular to the Y-axis that crosses through the radial styloid and the middle of the DRUJ was defined (Figure 2(c), blue line), which represents the Z-axis.
6. A line perpendicular to the Y- and Z-axis is defined, which represents the X-axis (Figure 2, red line).

In reference to the planes in a CT scan, the X-Y plane represents a coronal slice, the X-Z plane an axial slice and the Y-Z plane a sagittal slice. This coordinate system was used for both the affected and unaffected side.

### 3-D measurements

**Figure 3.**
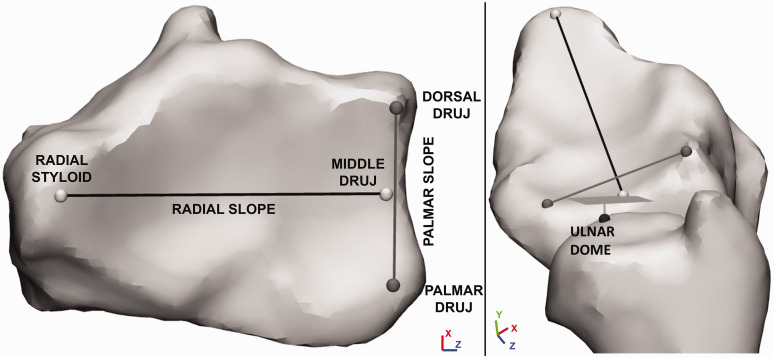
Relevant location identification on a 3-D model of the distal radius in the (a) axial and (b) medial view. The palmar and dorsal DRUJ points make up the palmar slope. The middle DRUJ point is determined as the halfway point between the palmar and dorsal DRUJ points. The middle DRUJ point and the radial styloid make up the radial slope line. The ulnar dome and the plane through the middle DRUJ point make up the ulnar variance. DRUJ: distal radioulnar joint.

Five relevant surface points were identified on the 3-D bone surface models of the affected radius and ulna: the radial styloid; the most palmar point of the DRUJ surface; the most dorsal point of the DRUJ surface; the point halfway between the palmar and dorsal DRUJ points; and the top of the ulnar dome (Figure 3). By connecting these surface points, two lines were drawn that were used to perform all 3-D measurements. The first line was drawn between the radial styloid and the middle DRUJ point, which represents the radial slope. The second line connected the palmar and dorsal DRUJ points and is called the palmar slope (Figure 3). Per side, 3-D RI was measured on the Y-Z plane as the angle between the radial slope and the Z-axis (Figure 1(d)). 3-D UV was measured as the distance in the Y-direction between the ulnar dome and the middle DRUJ point (Figure 1(e)). 3-D PT was measured on the X-Y plane as the angle between the palmar slope and the X-axis (Figure 1(f)).

AR was calculated bilaterally as the angle on the X-Z plane between the radial slope lines of both the unaffected and affected bone surface models (Figure 1(g)).

The 3-D measurements of RI, UV and PT were repeated on the unaffected side using the same protocol as described above, and the differences between the affected and unaffected wrists were calculated.

### Raters

All measurements on both the affected and unaffected side were performed by three raters: two technical physicians (CS and SvdH) and one 3-D specialized research scientist (EvE). The 2-D and 3-D measurements were performed separately, and raters were blinded from earlier measurements. One rater (CS) performed all measurements three times. Before the measurements, raters had a plenary session in which all measurements were performed on the data of one patient to reach consensus on measurement definitions. This patient was not included in the study.

For each patient, raters were given PA and lateral radiographs of the unaffected and affected sides and a 3-Matic file with the bone surface models. In 2-D, they performed RI, UV and PT measurements separately for both sides and stored images of the performed measurements. In 3-D, they constructed the coordinate system. Then, they identified the five relevant surface points per side. For each side separately, using these points, they constructed the lines, after which they performed the angle and distance measurements. The 3-Matic files with measurements were saved.

### Statistical analysis

The normality of the data was verified using the Shapiro–Wilk test and visually inspected using Q-Q plots. The mean and standard deviation for each parameter (2-D and 3-D: RI, UV, PT) were obtained. In inter-observer measurements, all raters had equal weights, meaning all intra-observer measurements together weighted equal to the measurements of other raters. The mean absolute differences between corresponding 2-D and 3-D measurements were calculated. In addition, the significance of differences between 2-D and 3-D RI, PT and UV were tested using paired samples *t*-tests (*p* < 0.05).

The reproducibility, through inter- and intra-observer variability, was investigated per measurement using an intraclass correlation coefficient (ICC). ICC values and their 95% confidence intervals were calculated based on a mean-rating (k = 3), absolute-agreement, two-way random-effects model. Inter-observer ICC values were calculated based on all three raters, and intra-observer ICC values were calculated on all three measurements of one rater. For the interpretation of the ICC values, the definition of Koo and Li (2016) was used, who define <0.5 as poor, 0.5 to <0.75 as moderate, 0.75 to <0.9 as good and 0.9 to 1.0 as excellent. The mean and standard deviation and inter- and intra-observer variability were likewise obtained for AR only in 3-D.

## Results

The mean age of the 35 included patients was 42 years (SD 18) and 28 were women. In total, 30 patients were right-handed, and 18 patients had a malunion of the dominant side. In 32 patients, standard radiographs of the unaffected side were available. In one patient, the lateral image was later deemed unusable. This patient was only included for RI and UV measurements.

### 3-D and 2-D values of affected and unaffected wrists

The 3-D and 2-D values of RI, UV and PT for affected and unaffected wrists and AR are presented in Table 1. The largest AR observed was 32.3°. For the affected side, we found mean differences between 3-D and 2-D of 1.8° (SD 1.5), 0.8 mm (SD 0.7) and 3.7° (SD 2.7) for RI, UV and PT, respectively. For the unaffected side, we found 1° (SD 1), 0.5 mm (SD 0.4) and 3.3° (SD 2.4), respectively (Table 1). The differences between 3-D and 2-D were found to differ significantly (*p* ≤ 0.01), except for PT in unaffected wrists (*p* = 0.63). All bilateral differences can be found in Appendix S1 as supplementary material online.

**Table 1.** Radial inclination, ulnar variance, palmar tilt and axial rotation measured in 35 patients with malunion of a distal radius fracture on 2-D wrist radiographs and 3-D CT.

	*n*	Mean (SD)	MAD (SD)	*p*
*Affected*				
RI (°)				
2-D	35	19.0 (5.4)	1.8 (1.5)	**0.009**
3-D	35	17.9 (6.2)		
UV (mm)				
2-D	35	2.3 (2.5)	0.8 (0.7)	**0.005**
3-D	35	2.8 (2.8)		
PT (°)				
2-D	34	−5.5 (14.1)	3.7 (2.7)	**0.011**
3-D	35	−7.4 (15.8)		
*Unaffected*				
RI (°)				
2-D	32	25.2 (2.8)	1.0 (1.0)	**0.012**
3-D	35	24.6 (2.3)		
UV (mm)				
2-D	32	0.3 (1.3)	0.5 (0.4)	**0.002**
3-D	35	0.0 (1.3)		
PT (°)				
2-D	31	13.3 (3.8)	3.3 (2.4)	0.633
3-D	35	12.9 (3.3)		
*Bilateral*				
AR (°)				
3-D	35	7.9 (6.9)		

Significant values (*p* < 0.05) are shown in bold.

AR: axial rotation; MAD: mean absolute difference; PT: palmar tilt; RI: radial inclination; UV: ulnar variance.

### Reproducibility

We found good to excellent inter-observer agreement (ICC 0.75–1) and excellent intra-observer agreement (ICC 0.9–1) for all but one measurement done in 2-D and all measurements done in 3-D. Only the 2-D PT measurements in the unaffected wrists showed a moderate inter- and intra-observer agreement (ICC 0.5–0.75). For all measurements, the ICC values were higher in 3-D compared to 2-D (Table 2). Statistically significant differences of inter-observer ICC were found for RI in affected wrists and for UV in unaffected wrists, and for intra-observer ICC in RI and PT in unaffected wrists. In addition, the reproducibility of AR measurements was excellent, with ICC values of >0.9 for both inter- and intra-observer measurements. ICC values of bilateral measurements can be found in Appendix S2 as supplementary material online.

**Table 2.** Inter- and intra-observer agreement values (with 95% CI) of RI, UV, PT and AR, measured on 2-D wrist radiographs and 3-D CT of 35 patients with malunion of a distal radius fracture.

	Inter-observer	Intra-observer
	ICC (95% CI)	ICC (95% CI)
*Affected*		
RI		
2-D	**0.94 (0.53 to 0.98)**	0.99 (0.98 to 1.00)
3-D	**0.99 (0.99 to 1.00)**	1.00 (0.99 to 1.00)
UV		
2-D	0.98 (0.94 to 0.99)	0.99 (0.97 to 1.00)
3-D	0.99 (0.99 to 1.00)	0.99 (0.98 to 1.00)
PT		
2-D	0.99 (0.98 to 0.99)	0.99 (0.98 to 1.00)
3-D	0.99 (0.98 to 0.99)	0.99 (0.99 to 1.00)
*Unaffected*		
RI		
2-D	0.82 (0.23 to 0.94)	**0.95 (0.87 to 0.98)**
3-D	0.94 (0.89 to 0.97)	**0.99 (0.99 to 1.00)**
UV		
2-D	**0.84 (0.64 to 0.93)**	0.93 (0.86 to 0.97)
3-D	**0.96 (0.93 to 0.98)**	0.97 (0.94 to 0.98)
PT		
2-D	0.60 (0.27 to 0.79)	**0.68 (0.38 to 0.84)**
3-D	0.80 (0.63 to 0.90)	**0.91 (0.84 to 0.95)**
*Bilateral*		
AR		
3-D	0.96 (0.92 to 0.98)	0.97 (0.95 to 0.99)

Significant different values between 2-D and 3-D (*p* < 0.05) are shown in bold.

AR: axial rotation; CI: confidence interval; PT: palmar tilt; RI: radial inclination; UV: ulnar variance.

## Discussion

In the present study, 3-D values were smaller than the corresponding 2-D values for all measurements, except for the UV measurement in affected wrists. Athlani et al. (2020) found comparable differences between 3-D and 2-D in affected dorsally tilted wrists, namely 3° for RI, 1.3 mm for UV and 2° for PT. Miyake et al. (2013) performed bilateral measurements and reported difference of 6° for RI, 1.8 mm for UV and 3° for PT between 3-D and 2-D. These are comparable to our bilateral measurements (Appendix S1).

We found that the inter- and intra-observer reproducibility was good or excellent for all measurements, and the ICC of 3-D measurements was higher than in 2-D. The reproducibility of measurements per side could not be compared with previous studies, since Athlani et al. (2020) did not report their reproducibility. However, Miyake et al. (2013) did report inter- and intra-observer reproducibility of their bilateral measurements. They found good or excellent ICC values for all measurements, in which reproducibility in 3-D was higher for UV and PT compared to 2-D but not for RI (see Appendix S2). Contrarily, we found that all 3-D measurements were more reproducible than in 2-D when performing bilateral measurements. Though reproducibility values of Miyake et al. (2013) are within the same range as found in our study, we found higher ICC values in all measurements compared to theirs, with the largest difference being 0.81 compared to 0.97 found in our study for inter-observer UV. We think this difference might be caused by the different measuring strategy that we used.

Furthermore, the 3-D measurements showed that AR was present in all patients, with a mean value of 7.9° (SD 6.9°) and with an excellent reproducibility. This is comparable to the mean AR reported by Athlani et al. (2020) of 9° (range 2°–21°) and smaller than the AR of Miyake et al. (2013), which was 13° (SD 11). The reproducibility of AR measurements was not reported in these studies.

The reproducibility of measurements (e.g. ICC) in the affected side was always higher than in the unaffected side. We believe this is caused by the way ICC values are calculated, because they are calculated as the ratio between the variance within raters and the total range of values within a variable (Bartko, 1966). Malunions cause a larger spread of the dataset in the affected side; hence, a similar variance within raters will result in higher ICC values. Due to differences in variances between measurements on the affected and unaffected sides, a comparison between the corresponding ICC values is invalid. Conversely, since corresponding 3-D and 2-D measurements have similar variances, comparisons between these measurements are valid.

In the present study, the ICC values of 2-D measurements were always lower than in 3-D, which could be the result of overlap of bony landmarks in 2-D radiographs, which complicates measurements. In 3-D, raters could view the anatomy in any direction, thereby decreasing the risk of misinterpretation of the anatomy. The larger inter-observer compared to intra-observer ICC between 3-D and 2-D measurements further strengthens this proposed explanation, as through a learning effect, because we expect that repeated measurements of one observer are less prone to this error.

Although the statistically significant differences between 2-D and 3-D are probably too small to be clinically relevant, we think that 3-D evaluation of malunion will become the standard. In our opinion, axial rotation might be an important factor in malunion evaluation, which is overlooked in 2-D measurements. Our expectation is that, once the clinical importance of axial rotation is proven, the shift towards routine 3-D evaluation of malunion may take place.

The strengths of our study are that by combining the knowledge from previous studies (Athlani et al., 2020; Miyake et al., 2013), we described a method that is well defined and easily performed provided similar software access. Using this method, 3-D measurements of malunion per side are obtained, which is usable in bilaterally affected patients as well. In addition, the construction of the 3-D coordinate system was in line with the ISB guidelines (Wu et al., 2005) and described explicitly, which encourages standardization in future research.

The present study has some limitations. First, all patients in this study had neutral or dorsally tilted extra-articular malunions. Therefore, it is uncertain whether our results apply to intra-articular or palmarly tilted malunions. Second, the unaffected side is currently used to obtain the coordinate system, which implies that taking a CT scan from the affected side only is not sufficient. Therefore, patients with bilateral affected wrists cannot be analysed this way. For these patients, a statistical shape model can be used (Mauler et al., 2017). Third, the intra-observer reproducibility was determined for only one observer. This could explain the higher ICC values, compared to the inter-observer measurements. Furthermore, a rater is more inclined to act upon non-normal measurements in unaffected wrists, compared to affected wrists, as they know normal values. This inherently causes mean differences between 3-D and 2-D values to be smaller in unaffected wrists. Finally, a general limitation of the use of a CT scan as opposed to plain radiograph images is the increased radiation dose. However, the mean effective radiation dose of a wrist CT scan is as low as 0.15 mSv (Iordache et al., 2017), due to constant improvements of CT scanning. In our opinion, the potential added benefit of 3-D analysis of the malunion using a CT scan outweighs the disadvantage of this small increase in radiation dose.

In conclusion, our study paves the way for routine 3-D analysis, planning and postoperative evaluation of extra-articular radius malunion parameters in affected and unaffected wrists.

## Supplemental Material

sj-pdf-1-jhs-10.1177_17531934231213790 - Supplemental material for A comparison of 3-D CT and 2-D plain radiograph measurements of the wrist in extra-articular malunited fractures of the distal radius Supplemental material, sj-pdf-1-jhs-10.1177_17531934231213790 for A comparison of 3-D CT and 2-D plain radiograph measurements of the wrist in extra-articular malunited fractures of the distal radius by Camiel J. Smees, Eline M. van Es, Gabriëlle J. M. Tuijthof, Joost W. Colaris, Feike de Graaff and Anne J. H. Vochteloo in Journal of Hand Surgery (European Volume)

sj-pdf-2-jhs-10.1177_17531934231213790 - Supplemental material for A comparison of 3-D CT and 2-D plain radiograph measurements of the wrist in extra-articular malunited fractures of the distal radius Supplemental material, sj-pdf-2-jhs-10.1177_17531934231213790 for A comparison of 3-D CT and 2-D plain radiograph measurements of the wrist in extra-articular malunited fractures of the distal radius by Camiel J. Smees, Eline M. van Es, Gabriëlle J. M. Tuijthof, Joost W. Colaris, Feike de Graaff and Anne J. H. Vochteloo in Journal of Hand Surgery (European Volume)
