# Supplementary material for: A comparison of 3-D CT and 2-D plain radiograph measurements of the wrist in extra-articular malunited fractures of the distal radius
Source: J Hand Surg Eur Vol. 2023 Nov 21;49(5):546–53. doi: 10.1177/17531934231213790 (PMC11044515; doi:10.1177/17531934231213790)
Supplement: sj-pdf-1-jhs-10.1177_17531934231213790 - Supplemental material for A comparison of 3-D CT and 2-D plain radiograph measurements of the wrist in extra-articular malunited fractures of the distal radius [file sj-pdf-1-jhs-10.1177_17531934231213790.pdf]

Table S1: Mean bilateral differences for radial inclination, ulnar variance, palmar tilt and axial rotation measured in 35 patients in this study, compared to 20 patients measured in the study of Miyake et al. (2013).

	This study		Miyake et al. (2013)	
Bilateral	Mean	MAD	Mean	MAD
RI (°)				
2D	6	2	15	6
3D	7		12	
UV (mm)				
2D	2.0	1.0	2.3	1.8
3D	2.8		3.0	
PT (°)				
2D	20	5	33	3
3D	21		30	
AR (°)				
3D	8		13	

RI: radial inclination; UV: ulnar variance; PT: palmar tilt; AR: axial rotation; MAD: mean absolute difference.
