# Supplementary material for: A comparison of 3-D CT and 2-D plain radiograph measurements of the wrist in extra-articular malunited fractures of the distal radius
Source: J Hand Surg Eur Vol. 2023 Nov 21;49(5):546–53. doi: 10.1177/17531934231213790 (PMC11044515; doi:10.1177/17531934231213790)
Supplement: sj-pdf-2-jhs-10.1177_17531934231213790 - Supplemental material for A comparison of 3-D CT and 2-D plain radiograph measurements of the wrist in extra-articular malunited fractures of the distal radius [file sj-pdf-2-jhs-10.1177_17531934231213790.pdf]

Table S2: Inter- and intraobserver correlation values for bilateral measurements of radial inclination, ulnar variance and palmar tilt measured in 35 patients in this study, compared to 20 patients in the study of Miyake et al. (2013)

	Interobserver ICC		Intraobserver ICC	
Bilateral	This study	Miyake et al.	This study	Miyake et al.
RI				
2D	0.97	0.96	0.99	0.95
3D	1.00	0.92	1.00	0.92
UV				
2D	0.97	0.81	0.98	0.90
3D	0.98	0.92	0.99	0.90
PT				
2D	0.94	0.90	0.99	0.95
3D	0.98	0.95	0.99	0.99

RI: radial inclination; UV: ulnar variance; PT: palmar tilt.
